# F24. OMEGA-6 PUFA METABOLISM DISTURBED IN PHOSPHO- AND SPHINGOLIPIDS IN NEUROLEPTIC-NAïVE FIRST-EPISODE SCHIZOPHRENIA PATIENTS – A FATTY ACID PROFILING STUDY IN FIVE LIPID FRACTIONS

**DOI:** 10.1093/schbul/sby017.555

**Published:** 2018-04-01

**Authors:** Kerstin Langbein, Christian Fleischer, Katrin Kuhnt, Stefan Smesny

**Affiliations:** 1 Jena University Hospital; 2 Friedrich-Schiller-University Jena

## Abstract

**Background:**

Alterations of polyunsaturated fatty acid (PUFA) levels are a well-replicated finding in schizophrenia research. There is however a controversy about the origin of this abnormality and its importance in the pathogenesis of schizophrenic illness. To investigate the influence of nutrition, in this study we investigate different aspects of fatty acid metabolization in a cohort of neuroleptic naïve first-episode schizophrenia patients (FEP) and a group of healthy controls (HC) matched for age and gender.

**Methods:**

In 33 FEP (25.8 ± 4.8y, male 60.6% 20/33) and 32 HC (24.9 ± 4.6y, male 53.1% 17/32) fatty acid profile was investigated by gas chromatography in blood plasma lipids of the triacylglycerol (TAG) fraction (closely related to fat consumption within recent days, rich in SFA, MUFA (~50%), and LA (C18:2n6)), the cholesterol ester (CE) fraction (dependent on fat consumption within recent days, rich in LA (C18:2n6)(~51%), SFA and PUFA), and the plasma phospholipid (PL) fraction (reflecting fat consumption of the last weeks to month, rich in SFA (~50%), AA (~7%), and LA (C18:2n6). In erythrocyte membranes (sensitive to fat consumption within weeks to month), fatty acid profile was investigated in phospholipids of the phosphoethanolamin (PE) fraction (rich in PUFA (~45%), AA (C20:4n6) and SFA) and of the sphingomyelin (SM) fraction (rich in long chain SFA (>70%) and MUFA (including NA C24:1c15). Psychopathology was assessed using the PANSS, BPRS-E and SCL-90-R ratings. Statistical analysis included multi- and univariate ANOVA, non-parametric tests and correlation analysis.

**Results:**

In the plasma PL fraction and in the lipid fractions of erythrocyte membranes (PE, SM) that are less influenced by recent nutrition, patients showed generally reduced omega-6 PUFA levels, particularly in terms of AA in the SM fraction. While PUFA of the PL and PE fraction were positively correlated in HC, this was not the case in the FEP group.

**Discussion:**

Our results support the previous finding of a general omega-6 PUFA deficit in FEP. The decrease points towards an endogenous regulation deficit that is independent of recent nutrition, might affect the metabolism of grey and white matter structural components, and could cause alterations of AA downstream functions. While correlation analysis in HC strongly suggests that nutrition and supplementation have the potential to influence PUFA availability, inner transport and metabolization pathways seem to be disturbed in FEP.

